# Development and validation of behavioral intention measures of an E-vapor product: intention to try, use, dual use, and switch

**DOI:** 10.1186/s12955-021-01764-2

**Published:** 2021-04-15

**Authors:** Stacey A. McCaffrey, Jessica Parker Zdinak, Stephanie Plunkett, Elizabeth Becker, Jennifer N. Lewis, Ryan A. Black

**Affiliations:** 1 JUUL Labs, Inc., 1000 F Street NW, Suite 800, Washington, DC 20004 USA; 2 Altria Client Services LLC, 601 East Jackson Street, Richmond, VA 23219 USA

**Keywords:** Behavioral intention, Psychometric validation, Rasch model, E-vapor, Intention to switch, Public health

## Abstract

**Background:**

The harm caused by tobacco use is primarily attributable to cigarette smoking. Switching completely to non-combustible products may reduce disease risks in adult cigarette smokers who are unable or unwilling to quit. Before a new tobacco product can enter the market or can be marketed as a modified risk tobacco product, the manufacturer must determine the impact that the product will have on the likelihood of changes in tobacco use behavior among both tobacco users and nonusers. One way to estimate change in tobacco use behavior is to assess tobacco users’ and nonusers’ *behavioral intentions* toward the product and its marketing, including intentions to try, use, dual use, and switch to the product from cigarettes. The purpose of this study was to develop and validate behavioral intention metrics appropriate for use with current, former, and never adult tobacco users.

**Methods:**

Preliminary items were subjected to cognitive testing with adult (1) smokers planning to quit cigarettes in the next 30 days, (2) smokers not planning to quit cigarettes in the next 30 days, (3) e-vapor users, (4) former tobacco users, and (5) never tobacco users. Items were iteratively revised based on feedback during cognitive testing, and surviving items were administered to a large sample of adults (N = 2943) representing the aforementioned sub-groups. Rating scale functioning, reliability, validity, bias, and ability to detect change were evaluated.

**Results:**

Examination of the response category thresholds generated by the Rasch model provided evidence that the rating scales were functioning appropriately. Results revealed good stability and excellent internal consistency and person reliability and provided evidence of unidimensionality and convergent validity. Estimates of reliability and validity were similar across sub-groups. A cross-validation sample generally confirmed findings from the validation sample. No items were discarded due to differential item function. Exploratory analyses provided support for ability to detect change.

**Conclusions:**

Results from this rigorous, empirical evaluation using large validation and cross-validation samples provide strong support for the psychometric properties of the Intention to Try, Use, Dual Use, and Switch scales with current, former, and never adult tobacco users.

**Supplementary Information:**

The online version contains supplementary material available at 10.1186/s12955-021-01764-2.

## Background

Most tobacco-related diseases are attributable to a cigarette smoker’s exposure to smoke. If adult smokers switch completely to non-combustible tobacco products, they may reduce their risk of smoking-related diseases. However, misperceptions hinder adult smoker adoption of potentially harm reducing non-combustible products. Adult smokers continue to have misperceptions regarding the role of nicotine and the relative risks of different tobacco products [1, 2]. For a manufacturer to sell a new, non-combustible tobacco product in the United States, it must be authorized by the Food and Drug Administration (FDA) through a manufacturer’s Premarket Tobacco Product Application (PMTA). To communicate that a non-combustible product may pose less risk than a combustible product, the FDA must determine that the product is a “modified risk tobacco product” using a manufacturer’s Modified Risk Tobacco Product Application (MRTPA). Both PMTAs and MRTPAs should include data on the effects of marketing and advertising on tobacco users’ and nonusers’ likelihood of trying and using the tobacco products. For example, the FDA’s Electronic Nicotine Delivery Systems (ENDS) PMTA Guidance recommends that applications incorporate evaluations of “use intentions among current ENDS users, nonusers, and other tobacco product users,” as well as the effects of the new tobacco product on users, “including effects on initiation, switching behavior, cessation, and dual use” [3]. Additionally, the FDA’s MRTPA Draft Guidance asks manufacturers to provide behavioral data to assess the likelihood that tobacco product users or nonusers will start using the modified risk product (i.e., trial and use) and that tobacco users will use the modified risk product “in conjunction with other tobacco products” (i.e., dual use) [4]. Lastly, from a public health perspective, there is an important distinction between trial and regular use of a product, as well as between dual use and switching, as these behaviors have different impacts on individual and population health.

Published literature, including a meta-analysis, has demonstrated an empirical causal relationship between intentions and behavior [5]; however, intentions should not be considered an exact proxy for behavior. The use of behavioral intentions to approximate and understand behavior in tobacco product research is consistent with FDA PMTA and MRTPA guidance [3, 4]. Moreover, collecting behavioral intentions data is necessary to provide evidence about new products and/or proposed modified risk communications that are not yet in the marketplace.

According to the FDA, validated items to capture behavioral intentions should be used in application submissions whenever possible [6]. Indeed, utilizing valid behavioral intention metrics is important for research conducted in support of PMTAs and MRTPAs, including research that explores the impact of modified risk messaging on behavioral intentions. However, at the present time a validated instrument to measure tobacco-related behavioral intentions does not exist in the published literature. Furthermore, in addition to having evidence of reliability and validity, the ideal tobacco-related behavioral intentions metric should be appropriate for use with adult tobacco users and nonusers, sensitive enough to detect change, and able to be used with various types of tobacco products.

Currently, there is substantial variability in the assessment of tobacco-related behavioral intentions across industry, academia, and government research. For example, some experimental and survey research studies utilize single-item behavioral intention scales [7, 8], other studies utilize discrete choice or product selection to infer intentions or likelihood of use [9, 10], and other studies capture more specific aspects of intentions, such as smoking expectation or interest, in hypothetical MRTPs [11]. Accordingly, our ability to compare results across studies and synthesize findings is limited when studies utilize different metrics (or correlates) of behavioral intentions. Having behavioral intention metrics with the idealistic qualities described above (e.g., reliability, validity, appropriate for tobacco users and nonusers, sensitive to detect change) may provide researchers in various contexts (academia, government, etc.) the opportunity to use a shared, psychometrically sound measurement tool.

The use of validated tobacco-related intentions metrics would directly address guidance from the FDA [3, 4] and help improve the quality of evidence gathered to support non-combustible tobacco product applications, including authorization of modified risk health communications to adult tobacco consumers. The authorized products and reduced harm communications ultimately play a critical role in moving smokers from cigarettes to noncombustible products.

### Current study

The current research represents the development of measures to assess behavioral intentions among tobacco users and nonusers following the rigorous validation process consistent with FDA guidance [12] and widely accepted standards [13, 14]. For example, research followed a multi-step process that is consistent with the FDA Patient-Reported Outcome (PRO) Guidance to support labeling claims [12]. Additionally, implementation of validated intention metrics, such as these behavioral intention measures, is consistent with recent FDA draft guidance [6]. Specifically, this study presents the development and validation of the Intention to Try (ITT), Intention to Use (ITU), Intention to Dual Use (ITDU), and Intention to Switch (ITS) scales referencing e-vapor products. Assessing a range of intentions (e.g., ITT, ITS) appears to be consistent with the FDA’s framework to understand the likelihood of initiation, repeated use, and conversion to lower risk tobacco products [15].

## Methods

The validation process employed in the current study included (1) development of the initial items, (2) item refinement based on cognitive interviews among tobacco users and nonusers and input from subject matter experts (SMEs), and (3) quantitative empirical evaluation.

A third-party vendor, Inflexxion, Inc. (Waltham, MA), collected and analyzed all data. A study protocol and supporting documents were submitted to Chesapeake IRB, reviewed under the Federal Policy for the Protection of Human Subjects (45 CFR Part 46), and the study was determined to be exempt from IRB oversight.

### Item development

The initial pool of items was developed by a team of SMEs (N = 8; representing the fields of cognitive, social, and clinical psychology; neuropsychology; psychometrics; and market research), drawing from published literature [16–18]. Published items were modified and new items were developed to adequately capture behavioral intentions toward an e-vapor product. Initial items incorporated key concepts from the literature such as degree of commitment or readiness (e.g., “I am open” vs. “I will”), perceptions of one’s social network (e.g., likelihood of trying a product if a friend offered the product to you), different levels of progress (e.g., “gradually” switching vs. planning to use as a “complete replacement”), and different temporal dimensions, including time until implementation (i.e., 30 days, 6 months) and frequency of use (i.e., “try,” “more than once,” “regularly use,” “will be my regular brand”). This initial draft survey was then subject to testing through individual semi-structured cognitive debriefing interviews.

### Cognitive interviews

#### Procedures

Cognitive interviewing is a qualitative research strategy used to improve the quality and accuracy of survey instruments. Through cognitive debriefing interviews, it is possible to identify and correct sources of response error in the survey and to verify that the survey (i.e., instructions, response options, items) is understood as intended, thereby enhancing and providing evidence of content validity [19, 20]. Retrospective probing techniques, which emphasize realism [19], were used in the current study.

Three experienced interviewers (two licensed clinical psychologists and a research assistant), trained on the semi-structured interviewing script and conducted in-person one-on-one interviews with participants over a three-day period at the Consumer Opinion Center (COC; https://www.consumeropinioncenter.com/) located in Virginia. Interviews were audio recorded to facilitate analysis, lasted approximately one hour, and participants were compensated $125 for their time.

After completing the informed consent, participants were asked to complete the survey independently while the interviewer observed, noting any potentially relevant behavioral observations (e.g., changing an answer). Next, using the semi-structured interviewing guide, the interviewer reviewed the survey item-by-item, including participants’ responses, with the participant. The interviewers utilized the general and specific probes from the interviewing guide, but also deviated as needed when signs of confusion, contradiction, or subtle misunderstandings were observed [19]. General probes included: *(1) How did you arrive at your answer? (2) Was there anything confusing about the question? (3) What do you think this question is asking?* consistent with general probing guidelines as described by Byrom and Tiplady [19]. Probes were intended to evaluate comprehension, retrieval, judgement, and response [19]. Examples of specific probes included: *What do you think they meant by “trying”? What do you think they mean by “expect to use” instead of “expect to try”? What do you think they mean by “smokes daily”? Why did you select [participant’s response] instead of [response option adjacent to participant’s response]?*

The three interviewers each interviewed 2–3 participants per day over a 3-day period. At the end of each day, interviewers met to discuss participant feedback, to identify themes (i.e., instances where multiple participants provided the same feedback or probing revealed a similar misunderstanding or opportunity for improvement), and to assess saturation. Once interviews were complete, results (themes, representative participant quotes) were compiled into a report and used to guide survey revisions.

#### Participants

Participants were recruited by the contract research organization Celerion Inc. (Lincoln, NE) through the COC. Celerion recruited, contacted, and screened potentially eligible adults through their database and other recruitment methods (fliers, other advertisement materials) for inclusion in the study. To qualify for participation, participants had to: (1) be of legal age to purchase tobacco or older, whether or not they were a current user of tobacco, in the state and locality in which they resided, (2) provide voluntary consent, (3) acknowledge willingness and ability to comply with all study requirements, and (4) meet criteria for inclusion in one of the five study sub-groups. These study sub-groups included: adult (1) cigarette smokers planning to quit cigarettes in the next 30 days (ASPQ), (2) cigarette smokers not planning to quit in the next 30 days (ASNPQ), (3) e-vapor users (EV Users), (4) former tobacco users (Former Users), and (5) never tobacco users (Never Users).

### Empirical evaluation

#### Participants

Participants were recruited from a nationally representative online community of panelists who provided political and demographic information about themselves through PollingPoint, which was owned and operated by YouGov (London, UK; https://today.yougov.com/). Interested YouGov members completed a brief screener to determine eligibility. Inclusion criteria for this part of the study were the same inclusion criteria used during cognitive interviewing. Eligible participants who provided consent completed the electronic survey through the YouGov online survey platform. To ensure the findings obtained from the empirical evaluation were stable over sampling and generalizable across sub-groups, the survey was open until approximately 600 individuals per sub-group had participated.

Three days after completing the survey, a sub-sample of participants was re-contacted to complete the survey again to gather information about the items’ stability. Invitations were sent to members on a rolling basis after completing the initial survey until a minimum of 100 participants per sub-group had completed the retest. The sample size of *n* = 100 was derived from a power analysis to detect a significant difference between an intraclass correlation coefficient (ICC) of 0.80 (within the acceptable level) and 0.69 (below the acceptable level), assuming 80% power.

#### Measures

Participants completed an electronic survey that included questions about demographics, tobacco use history and current tobacco use, and the behavioral intention items. Before exposure to the behavioral intention items, participants were provided with a brief description of the e-vapor products, as well as the instructions: *Please rate the extent to which you agree or disagree with the following items. We realize you may not know the answer to each question, but please provide your best answer. Please refer to the description [of the product] above to help you answer the following items.*

Participants responded to the behavioral intention items shown in Table 3. Multi-item scales (ITT, ITU, ITS) were scored by calculating a mean of the items within each scale.

After answering the behavioral intention items, participants responded to a Behavioral Selection task, adapted from previous research [21]: *If we have the opportunity to send you one of the products listed below for free, which of the products would you choose? One of the [specific brand] e-vapor products / another e-vapor product not shown / gas card of similar value as an e-vapor product / I would not wish to receive any of these.* For purposes of evaluating convergent validity and ability to detect change, the Behavioral Selection task was collapsed into a binary variable to reflect those who selected *One of the [specific brand] e-vapor products* vs. those who made an alternative response selection.

#### Analytic plan

The empirical evaluation was an iterative process, which included both modern test theory and classical test theory (CTT) approaches. First, a Rasch partial credit model [22] was employed in WINSTEPS analysis software to evaluate rating scale functioning. An important assumption underlying the use of a Likert-type rating scale is monotonicity, i.e., it requires greater intention to endorse a higher (more severe) response option (e.g., *Agree* vs. *Strongly Agree*). This assumption was empirically evaluated by examining the order of the response option Andrich thresholds estimated by the Rasch model, where response option Andrich thresholds are defined as the trait level at which a respondent has an equal probability of endorsing adjacent categories [23].

Second, unidimensionality, adequate item fit, and item discrimination were evaluated. Unidimensionality was evaluated in three ways:

1. In WINSTEPS, unidimensionality was evaluated by conducting a principal component analysis (PCA) on the probability scale residuals estimated from the Rasch model [24]. Additionally, factor sensitivity ratios [25] were calculated by dividing the residual variance eigenvalue units by the Rasch measure variance eigenvalue units [26, 27];
2. Monte Carlo simulations (“parallel analyses”) of 10,000 randomly generated parallel datasets were conducted to determine the number of significant factors derived from the PCA [28]. The eigenvalues derived from the PCA were compared against the 95th percentile of the distribution of the randomly generated eigenvalues to determine the number of significant factors;
3. a one-factor, first-order confirmatory factor analysis was employed to confirm the unidimensional structure of the scales using the cross-validation sample data in AMOS.

Item fit and item discrimination were evaluated by examining inlier and outlier item mean squares and discrimination statistics generated from WINSTEPS.

Third, to evaluate reliability, person reliability coefficients were generated from WINSTEPS. Additionally, internal consistency reliability was estimated using Cronbach’s α and test–retest reliability was captured by an ICC using absolute agreement.

Fourth, convergent validity was established by examining the relationship between behavioral intentions and selection of *one of the [specific brand] e-vapor products* on the Behavioral Selection task by employing Pearson correlations. Significant positive correlations were anticipated between intentions and selection of *one of the [specific brand] e-vapor products* on the Behavioral Selection task.

Fifth, bias with respect to gender, race (White/non-White), age (legal age to 24 years vs. > 24 years), and sub-group membership was evaluated via differential item function (DIF) [29]. An item was considered to have little or no difference between groups if the DIF Mantel–Haenszel contrast estimate was < 1 in absolute value and the *p*-value was non-significant [30, 31]. Of note, bias was evaluated for young adults (legal age to 24 years) compared to older adults because young adults are a population of interest to the FDA [32]. Bias was evaluated using the full sample to ensure that the sample sizes were large enough to achieve stable estimates.

Finally, we explored the scales’ ability to detect change in behavioral intentions over time. That is, while little to no true change in behavioral intentions was expected to occur during the three-day interval of time between administrations, any change would be expected to correspond with change in participants’ selection of *one of the [specific brand] e-vapor products* on the Behavioral Selection task. Ability to detect change was estimated by correlating residualized change scores [33] between the intentions scales and the Behavioral Selection task captured during the first and second test administrations.

Internal structure, internal consistency reliability, test–retest reliability, and convergent validity were evaluated across the five study sub-groups. Analyses were confirmed using a cross-validation sample.

Analyses were conducted using WINSTEPS version 3.74.0 [34], SPSS version 20 [35], and AMOS version 20 [36].

## Results

### Cognitive interviews

In total, 23 cognitive interviews were completed. The mean age of participants was 43.7 years (standard deviation [SD] = 12.3), and the majority of participants identified as male (73.9%). Approximately half of the participants reported full-time employment, and most participants had obtained a high school diploma/GED (39.1%) or completed some college (43.5%).

Items were iteratively removed or revised as appropriate based on themes identified from cognitive interviews in conjunction with SME input. For example, for the ITU measures, the original 5-point rating scale included a middle category "Neither Agree or Disagree." However, cognitive testing revealed that participants were interpreting and utilizing this category in meaningfully different ways (i.e., "I do not have an opinion," "I have mixed opinions"), threatening the validity of the response scale and increasing measurement error. Therefore, "Neither Agree or Disagree" was replaced with "Somewhat agree" and "Somewhat disagree," resulting in a 6-point scale. Additionally, prior to the empirical evaluation, SMEs made final selections regarding which items from the pool would be retained for purposes of capturing ITT, ITU, ITDU, and ITS in an effort to reduce scale length and respondent burden. Surviving items were subject to empirical evaluation.

### Empirical evaluation

#### Participants

Of the 40,604 participants who completed the screening, 32,488 did not meet inclusion criteria, 5173 provided incomplete or unusable data (i.e., completed the full survey in the top 2% of fastest times [under 5 min]), and 2943 completed the survey (full sample). Demographic characteristics for the full sample (N = 2943) and five sub-groups are presented as an additional file (see Additional file 1). Of the 2943 participants, 562 completed the second administration of the survey (ASPQ *n* = 101, ASNPQ *n* = 107, EV Users *n* = 104, Former Users *n* = 118, Never Users *n* = 132). The full sample was randomly split into validation (*n* = 1495) and cross-validation (*n* = 1448) samples. Participant demographic characteristics were similar across the five sub-groups, as well as between the validation and cross-validation samples (see Tables 1 and 2).

**Table 1:** Participant demographic characteristics across the validation sample and 5 sub-groups

Demographic characteristic	Validation sample (N = 1495)	ASPQ (n = 260)	ASNPQ (n = 333)	EV Users (n = 277)	Former Users (n = 302)	Never Users (n = 323)
Gender %						
Female	57.1	53.1	59.8	60.3	51.3	60.1
Male	42.9	46.9	40.2	39.7	48.7	39.9
Ethnicity %						
Non-Hispanic	93.4	93.8	95.8	92.4	92.4	92.6
Hispanic	6.5	6.2	4.2	7.6	7.3	7.4
Race %						
White/Caucasian	86.6	83.1	89.2	88.8	88.7	83.0
Black/African American	8.8	12.7	6.6	8.7	7.0	9.6
Asian	2.4	2.3	1.8	2.2	0.7	5.0
Native Hawaiian/Pacific Islander	0.5	0.4	0.3	0.7	0.3	0.6
American Indian/Alaska Native	2.3	2.7	2.1	2.9	2.3	1.5
Other	2.8	1.9	2.4	3.2	3.0	3.4
Region %						
Northeast	22.9	21.5	24.3	26.0	19.9	22.9
Midwest	22.1	21.2	24.9	20.9	21.5	21.7
South	31.4	36.2	30.6	26.7	32.5	31.6
West	22.7	20.0	19.8	25.6	25.2	23.2
Age						
Mean (SD)	52.0 (14.1)	51.1 (13.3)	51.4 (12.6)	47.1 (13.4)	57.4 (13.9)	52.7 (15.4)
Range (years)	18–90	21–82	22–82	18–78	19–88	19–90

Summary of participant demographic characteristics for participants in the full validation sample, as well as for the five study sub-groups. Raw percentages are reported; therefore, percentages do not always add to 100% due to missing data

ASPQ, adult smoker planning to quit; ASNPQ, adult smoker not planning to quit; EV, e-vapor

**Table 2:** Participant demographic characteristics across the cross-validation sample and 5 sub-groups

Demographic characteristic	Cross-validation sample (N = 1448)	ASPQ (n = 248)	ASNPQ (n = 315)	EV Users (n = 283)	Former Users (n = 275)	Never Users (n = 327)
Gender %						
Female	55.2	56.0	61.0	58.0	49.1	52.0
Male	44.8	44.0	39.0	42.0	50.9	48.0
Ethnicity %						
Non-Hispanic	91.8	91.5	94.3	89.4	91.6	91.7
Hispanic	8.1	8.5	5.4	10.6	8.4	8.0
Race %						
White/Caucasian	85.8	86.3	85.1	87.6	88.0	82.6
Black/African American	8.2	9.3	8.9	5.7	8.4	8.9
Asian	2.4	2.0	1.6	3.9	1.5	3.1
Native Hawaiian/Pacific Islander	0.5	0.4	0.6	1.1	0.0	0.3
American Indian/Alaska Native	2.9	3.6	3.5	3.9	2.5	1.2
Other	4.0	4.4	3.8	4.2	3.3	4.3
Region %						
Northeast	22.9	28.6	20.0	21.2	22.2	23.5
Midwest	21.1	17.7	22.2	20.1	24.4	20.5
South	32.1	29.8	37.8	31.4	29.8	30.9
West	23.7	23.8	20.0	26.5	23.6	24.8
Age						
Mean (SD)	53.0 (14.1)	51.6 (13.5)	51.7 (12.7)	49.6 (13.5)	58.6 (14.2)	53.5 (15.2)
Range (years)	18–88	22–83	23–85	21–77	18–88	18–86

Summary of participant demographic characteristics for participants in the full cross-validation sample, as well as for the five study sub-groups. Raw percentages are reported; therefore, percentages do not always add to 100% due to missing data

ASPQ, adult smoker planning to quit; ASNPQ, adult smoker not planning to quit; EV, e-vapor

Of note, as the ITDU scale consisted of a single item, only test–retest reliability and convergent validity were evaluated. The final behavioral intention items and their rating scales are presented in Table 3.

**Table 3:** Behavioral intention item content

Scale		Item content
ITT	Try1	I am open to trying [specific brand] e-vapor products in the next 30 days
	Try2	Based on what you know about [specific brand] e-vapor products, how likely or unlikely are you…? to try a [e-vapor product]
	Try3	Based on what you know about [specific brand] e-vapor products, how likely or unlikely are you…? to try a [specific brand] e-vapor product if one of your best friends were to offer a [specific brand] e-vapor product to you
ITU	Use1	I would consider using a [specific brand] e-vapor product more than once
	Use2	I expect to use a [specific brand] e-vapor product
	Use3	It is likely that I will regularly use a [specific brand] e-vapor product in the next 6 months
	Use4	A [specific brand] e-vapor product will be my regular brand of e-vapor/e-cigarette in the next 30 days
ITDU	DualUse1	I plan to use one of the [specific brand] e-vapor products in addition to regular cigarettes
ITS	Switch1	I plan to gradually switch from regular cigarettes to a [specific brand] e-vapor product
	Switch2	I plan on using one of the [specific brand] e-vapor products as a complete replacement for regular cigarettes
	Switch3	I intend on switching from cigarettes to a [specific brand] e-vapor product in the next 6 months

Item content for the behavioral intention items tested empirically as part of the current study. All items utilized the same 6-point fully labeled rating scale (1 = Strongly disagree, 2 = Disagree, 3 = Somewhat disagree, 4 = Somewhat agree, 5 = Agree, 6 = Strongly agree) except for Try2 and Try3, which utilized a 6-point likelihood scale (1 = Definitely not, 2 = Very unlikely, 3 = Somewhat unlikely, 4 = Somewhat likely, 5 = Very likely, 6 = Definitely)

#### Rating scale functioning

Evaluation of rating scale performance revealed ordered thresholds, suggesting that a higher level of intention is required to endorse a greater level of agreement or likelihood. To illustrate, response category thresholds among all participants in the validation sample are presented in Table 4.

**Table 4:** Response category thresholds

Item	Threshold 1	Threshold 2	Threshold 3	Threshold 4	Threshold 5
Try 1	− 4.70	− 1.64	-.62	2.05	4.91
Try 2	− 4.97	− 2.68	-.85	2.75	5.74
Try 3	− 4.00	− 2.04	-1.03	2.26	4.80
Use 1	− 7.46	− 2.55	-.57	3.71	6.87
Use 2	− 6.89	− 3.09	.24	3.44	6.30
Use 3	− 6.11	− 3.08	.16	3.23	5.81
Use 4	− 6.12	− 3.22	.25	3.17	5.91
Switch 1	− 10.85	− 3.53	.74	5.13	8.51
Switch 2	− 9.63	− 3.37	.57	4.89	7.54
Switch 3	− 9.03	− 3.43	.55	4.64	7.27

This table presents the category response thresholds for each of the ITT, ITU, and ITS items using data from the validation sample. The thresholds for each item are sequentially ordered from lowest to highest, providing empirical support for rating scale functioning

#### Model assumptions

The PCA on the probability scale residuals revealed that the Rasch model explained 81.2%, 84.0%, and 85.9% of the raw variance in the ITT, ITU, and ITS scales. Additionally, factor sensitivity ratios [25] for the ITT, ITU, and ITS scales were 13.3%, 8.4%, and 8.6%, respectively, suggesting the absence of multiple dimensions [26, 27].

Using the validation sample data, parallel analyses were conducted with each of the five sub-groups for each intention scale. The eigenvalues associated with the first factor was the only significant eigenvalue, providing support for unidimensionality of the scales with all five sub-groups. Finally, factor loading estimates and goodness-of-fit indices from the confirmatory factor analyses using the cross-validation sample provided additional support for unidimensionality (Table 5).

**Table 5:** Standardized loadings and fit indices of the unidimensional confirmatory factor analytic models

		Item	Loading	Chi-Square		CFI	GFI	RMSEA
				Statistic	*p*			
ITT^a^	Full cross-validation sample	Try1	.940	11.724	.001	.998	.995	.086
		Try2	.966					
		Try3	.940					
	ASPQ	Try1	.926	.056	.813	1.000	1.000	.000
		Try2	.962					
		Try3	.917					
	ASNPQ	Try1	.874	5.015	.025	.995	.990	.113
		Try2	.944					
		Try3	.875					
	EV Users	Try1	.853	1.841	.175	.998	.996	.055
		Try2	.912					
		Try3	.831					
	Former Users	Try1	.902	.081	.775	1.000	1.000	.000
		Try2	.959					
		Try3	.971					
	Never Users	Try1	.860	.084	.772	1.000	1.000	.000
		Try2	.969					
		Try3	.973					
ITU^b^	Full cross-validation sample	Use1	.927	21.910	.000	.998	.993	.120
		Use2	.996					
		Use3	.945					
		Use4	.915					
	ASPQ	Use1	.862	.538	.463	1.000	.999	.000
		Use2	.933					
		Use3	.946					
		Use4	.968					
	ASNPQ	Use1	.845	1.485	.223	1.000	.998	.039
		Use2	.998					
		Use3	.888					
		Use4	.792					
	EV Users	Use1	.817	8.856	.003	.994	.985	.167
		Use2	.935					
		Use3	.969					
		Use4	.907					
	Former Users	Use1	.912	.055	.815	1.000	1.000	.000
		Use2	.984					
		Use3	.990					
		Use4	.983					
	Never Users	Use1	.929	12.863	.000	.995	.981	.191
		Use2	.989					
		Use3	.971					
		Use4	.956					
ITS^a^	Full cross-validation sample	Switch1	.958	.006	.938	1.000	1.000	.000
		Switch2	.963					
		Switch3	.961					
	ASPQ	Switch1	.957	.784	.376	1.000	.998	.000
		Switch2	.975					
		Switch3	.973					
	ASNPQ	Switch1	.953	.332	.564	1.000	.999	.000
		Switch2	.954					
		Switch3	.934					
	EV Users	Switch1	.942	.076	.783	1.000	1.000	.000
		Switch2	.945					
		Switch3	.960					

Standardized loadings and fit indices of the unidimensional confirmatory factor analytic models for the multi-item Intention scales among participants in the cross-validation sample

ASPQ, Adult Smokers Planning to Quit; ASNPQ, Adult Smokers Not Planning to Quit; EV, e-vapor; CFI, comparative fix index; GFI, goodness of fit index; RMSEA, root mean square error of approximation

^a^To achieve over-identification, two error variances similar in magnitude were constrained to be equal

^b^Due to shared item content and to improve model fit, the error covariance between items was freely estimated

All items evidenced adequate fit statistics (both infit and outfit values were not > 1.50) [27], suggesting that the items were functioning as expected for the Rasch model. Moreover, item discrimination values, estimated outside of the Rasch model, produced good fit to the model (approximately 0.5 to 1.7) [37].

#### Reliability

Person reliability coefficients (derived from the Rasch model) for the ITT, ITU, and ITS scales were 0.87, 0.92, and 0.90, respectively, providing evidence that the scales are able to accurately quantify persons with different levels of intention. Internal consistency reliability estimates were consistently high across subgroups (Cronbach’s α = 0.876—0.985) (Table 6). Test–retest reliability coefficients were largely moderate to good [38] (absolute ICC = 0.650—0.858), except for the reliability coefficient for ITDU among EV Users (absolute ICC = 0.395). The substantial discrepancy between the validation and cross-validation sample ITDU stability coefficients for EV Users suggests that the sample sizes were too small to yield stable estimates (*n* = 24). Therefore test–retest reliability for ITDU among EV Users was recalculated using the full sample (*n* = 48; absolute ICC = 0.544, *p* < 0.001).

**Table 6:** Internal consistency and test–retest reliability

Scale	Sub-Group	Validation sample				Cross-validation sample			
		Test–retest reliability		Internal consistency reliability		Test–retest reliability		Internal consistency reliability	
		n	ICC (*p)*	n	α	n	ICC (*p)*	n	α
ITT	Full sample	279	.886 (< .001)	1495	.958	283	.930 (< .001)	1448	.962
	ASPQ	48	.790 (< .001)	260	.938	53	.806 (< .001)	248	.953
	ASNPQ	58	.745 (< .001)	333	.923	49	.929 (< .001)	315	.922
	EV Users	54	.650 (< .001)	277	.876	50	.885 (< .001)	283	.899
	Former Users	57	.672 (< .001)	302	.948	61	.893 (< .001)	275	.957
	Never Users	62	.858 (< .001)	323	.945	70	.861 (< .001)	327	.950
ITU	Full sample	279	.886 (< .001)	1495	.973	283	.911 (< .001)	1448	.973
	ASPQ	48	.838 (< .001)	260	.968	53	.781 (< .001)	248	.966
	ASNPQ	58	.761 (< .001)	333	.946	49	.910 (< .001)	315	.939
	EV Users	54	.796 (< .001)	277	.935	50	.862 (< .001)	283	.952
	Former Users	57	.715 (< .001)	302	.972	61	.816 (< .001)	275	.983
	Never Users	62	.741 (< .001)	323	.985	70	.908 (< .001)	327	.978
ITDU	Full sample	115	.759 (< .001)	–	–	109	.798 (< .001)	–	–
	ASPQ	42	.817 (< .001)	–	–	46	.721 (< .001)	–	–
	ASNPQ	49	.742 (< .001)	–	–	39	.684 (< .001)	–	–
	EV Users^a^	24	.395 (.026)	–	–	24	.755 (< .001)	–	–
ITS	Full sample	115	.813 (< .001)	742	.965	109	.834 (< .001)	701	.973
	ASPQ	42	.858 (< .001)	260	.972	46	.746 (< .001)	248	.978
	ASNPQ	49	.687 (< .001)	333	.964	39	.880 (< .001)	315	.963
	EV Users	24	.739 (< .001)	149	.920	24	.857 (< .001)	138	.964

Reliability coefficients for the Intention scales among the full validation and cross-validation samples, as well as among the 5 study sub-groups

ASPQ, Adult Smokers Planning to Quit; ASNPQ, Adult Smokers Not Planning to Quit; EV, e-vapor; ICC, Intraclass correlation coefficient

^a^Test retest reliability among the full sample (n = 48) was ICC = .544, *p* < .001

Aside from the aforementioned exception, results using the cross-validation sample were generally consistent with findings using the validation sample.

#### Convergent Validity

As evidence of convergent validity, there was a significant positive association between intentions and selection of *one of the [specific brand] e-vapor products* on the Behavioral Selection task (Table 7). Results from the cross-validation sample confirmed this finding.

**Table 7:** Convergent validity coefficients

Scale	Sub-group	Validation sample		Cross-validation sample	
		Behavioral selection		Behavioral selection	
		n	*r* (*p*)	n	*r* (*p*)
ITT	All sub-groups	1495	.694 (< .001)	1448	.744 (< .001)
	ASPQ	260	.560 (< .001)	248	.656 (< .001)
	ASNPQ	333	.557 (< .001)	315	.573 (< .001)
	EV Users	277	.472 (< .001)	283	.562 (< .001)
	Former Users	302	.571 (< .001)	275	.678 (< .001)
	Never Users	323	.644 (< .001)	327	.627 (< .001)
ITU	All sub-groups	1495	.681 (< .001)	1448	.722 (< .001)
	ASPQ	260	.565 (< .001)	248	.667 (< .001)
	ASNPQ	333	.560 (< .001)	315	.541 (< .001)
	EV Users	277	.436 (< .001)	283	.498 (< .001)
	Former Users	302	.534 (< .001)	275	.682 (< .001)
	Never Users	323	.675 (< .001)	327	.622 (< .001)
ITDU	All sub-groups	742	.451 (< .001)	701	.551 (< .001)
	ASPQ	260	.391 (< .001)	248	.490 (< .001)
	ASNPQ	333	.514 (< .001)	315	.525 (< .001)
	EV Users	149	.165 (.044)	138	.308 (< .001)
ITS	All sub-groups	742	.537 (< .001)	701	.584 (< .001)
	ASPQ	260	.604 (< .001)	248	.669 (< .001)
	ASNPQ	333	.524 (< .001)	315	.513 (< .001)
	EV Users	149	.213 (.009)	138	.360 (< .001)

Pearson correlations between the Intention scales and the Behavioral Selection task among the full validation and cross-validation samples, as well as among the 5 study sub-groups

ASPQ, Adult Smokers Planning to Quit; ASNPQ, Adult Smokers Not Planning to Quit; EV, e-vapor; ICC, Intraclass correlation coefficient

#### Bias

The ITT and ITS items did not exhibit substantial DIF for gender, race, age, or sub-group membership. While the ITU items did not exhibit substantial DIF for gender, race, or age, of the 40 between sub-group DIF comparisons, five emerged as significant. That is, “Use1” exhibited some evidence of DIF between the Never User group and the ASPQ, ASNPQ, and EV User groups, and “Use4” exhibited some evidence of DIF between the Never User group and the ASPQ and ASNPQ groups. However, these DIF contrasts were in the opposite direction (e.g., Use1 was more difficult for Never Users to endorse, and Use4 was easier for Never Users to endorse), suggesting that DIF is broadly cancelled out when an ITU composite is calculated [39]. Consequently, no items were discarded due to DIF.

#### Ability to detect change

Change in intention scores over a three-day test–retest period corresponded with change in selection of *one of the [specific brand] e-vapor products* on the Behavioral Selection task, evidenced by positive associations between residualized change scores (see Table 8). That is, participants who did not select *one of the [specific brand] e-vapor products* at the first Behavioral Selection task administration and then later selected *one of the [specific brand] e-vapor products* at the second task administration also reported an increase in behavioral intentions.

**Table 8:** Ability to detect change

Scale	Validation sample		Cross-validation sample	
	n	Pearson r (*p*)	n	Pearson r (*p*)
ITT	279	.217 (< .001)	283	.200 (< .001)
ITU	279	.322 (< .001)	283	.170 (.004)
ITDU	115	.093 (.322)	109	.227 (.018)
ITS	115	.421 (< .001)	109	.094 (.330)

This table provides the Pearson correlations between residualized change scores on the behavioral intention scale and the Behavioral Selection task, as measured at survey administration 1 and 2 (completed approximately three days apart)

#### Administration and scoring

The ITT, ITU, ITDU, and ITS scales were empirically validated in electronic form. Therefore, the extent to which the psychometric properties obtained through the current empirical validation generalize to a paper-and-pencil form is unknown. As such, electronic administration of the behavioral intention scales is recommended. The scales are scored by calculating a mean of the items within that scale. If response to an item is missing, a composite for that construct should not be calculated.

## Discussion

This is the first scientific publication to date describing the successful development and validation of instruments to measure tobacco-related behavioral intentions, including ITT (n = 3 items), ITU (n = 4 items), ITDU (n = 1 item), and ITS (n = 3 items) scales. Results from these behavioral measures can provide evidence to the FDA supporting PMTAs and MRTPAs on how adult smokers might use a new, non-combustible product or a non-combustible product with a reduced harm claim. Rasch modeling was employed to evaluate rating scale functioning and results suggested that the 6-point rating scales were functioning as expected. Multi-item scales were found to be unidimensional, and the scales evidenced excellent internal consistency and person reliability and good stability. Results provided support for convergent validity and exploratory analyses suggested that the scales may be able to detect true change over time. Lastly, none of the items exhibited significant DIF based on gender, race, or age. Although there was some evidence of DIF between sub-groups for two of the ITU items, these differences were considered inconsequential when computing composite scores.

It is noteworthy that estimates of internal structure, internal consistency reliability, test–retest reliability, and convergent validity were largely consistent across the five sub-groups. Taken together, the results suggest that the behavioral intention scales can be used across these different populations and direct comparisons between populations can be made without modifying scoring. The findings were robust as evidenced by similar results across validation and cross-validation samples. Results from this comprehensive evaluation also provide evidence that the Intention scales are appropriate for use among adult tobacco users, former users, and never users. This study helps support research conducted for tobacco regulatory applications by providing a valid measure of behavioral intentions, which is one important factor to consider in the overall assessment of tobacco use behavior.

The success of these scales may be partially due to the reliance on cognitive testing for item development, where qualitative feedback was obtained from a diverse group of participants with different types of experience with tobacco products. Qualitative testing likely improved the clarity of the scales (including the instructions, item content, and response options) and increased the content validity of the items. Additionally, the large sample size utilized in the empirical evaluation allowed for assessment of item functioning across sub-groups of participants. Separate evaluation of scale functioning across groups of tobacco users and nonusers is important to ensure adequate and similar psychometric functioning between groups.

In the current study, the behavioral intention items were validated in reference to a specific e-vapor brand. Recent research has also demonstrated that these intention scales are valid when modified to reference other tobacco and/or nicotine products, namely, an oral product containing tobacco-derived nicotine and a moist snuff tobacco product [40]. Specifically, the results of this research suggest that (1) the scales are reliable and valid when modified to reference other tobacco products, and (2) the intention scales function similarly (i.e., do not exhibit substantial DIF) across tobacco products.

For smokers to realize reduced risk of certain smoking-related diseases from non-combustible products, they must completely switch from cigarettes to the non-combustible product. The results presented here offer successful validation of behavioral intention measures that researchers and the public health community can use to better evaluate the impact of introducing a potentially reduced risk product or product with a reduced harm claim into the marketplace.

### Limitations and future research

The stability of the ITDU item over the three-day test–retest period was lower than anticipated for the EV User group. This finding may reflect greater fluctuation in e-vapor users’ true intention to dual use different e-vapor products. However, it should also be noted that absolute agreement in participants’ responses to the dual use item (ICC with absolute agreement) reflects a rather conservative estimate of scale reliability [38].

Cognitive testing relied on a convenience sample of participants from the Richmond, Virginia area. Inclusion of participants from other geographic locations may have improved wording of the items for use with individuals from other areas of the country. However, it should be noted that the sample from the Richmond location consisted of diverse groups of individuals across key demographic variables, including age, gender, and race as well as tobacco use history. The online sampling for the quantitative evaluation was devised to obtain a demographic distribution reflective of the US population from a nationally representative panel through PollingPoint, formerly of YouGov.

This research was conducted using adult (legal age to use tobacco and older) tobacco users and nonusers. Therefore, the extent to which these items are appropriate to capture behavioral intentions in youth cannot be inferred from this study.

An important next avenue of research will be to evaluate the predictive validity of the behavioral intention scales through longitudinal research that captures actual behavior. Evaluating the relationship between responses to these behavioral intention items and actual behavior would facilitate the development of cut points. Future research might also evaluate the scales’ ability to detect change over a longer period of time.

Despite the stated limitations, results from this evaluation provide support for the psychometric properties of the ITT, ITU, ITDU, and ITS scales specifying an e-vapor product with current, former, and never adult tobacco users.

## Conclusions

For new, non-combustible products or reduced harm claims to be authorized by the FDA, they must be supported by research demonstrating adult tobacco users’ and nonusers’ intentions to try and use, and adult tobacco users’ intentions to dual use or switch to the products. This study presents the first development and validation of behavioral intention scales appropriate for research studies supporting PMTAs and MRTPAs. Results from a comprehensive empirical evaluation provide evidence of reliability and validity of the ITT, ITU, ITDU, and ITS scales with current, former, and never adult tobacco users. Given the scales’ strong psychometric properties, these behavioral intention metrics may be used to capture changes in intention following exposure to modified risk messaging, marketing, and/or advertising.

## Supplementary Information

**Additional file 1**. Participant Demographic Characteristics Across the Full Sample and 5 Sub-groups. Description of data: Summary of participant demographic characteristics for the full study sample, as well as for the five study sub-groups.

## Data Availability

Reasonable requests for datasets and/or analyses presented in this manuscript will be considered as appropriate, recognizing that the data is currently subject to review as part of a proprietary product application pending with FDA.

## Abbreviations

ASNPQ: Adult smoker not planning to quit
ASPQ: Adult smoker planning to quit
CFI: Comparative fix index
COC: Consumer Opinion Center
CTT: Classical test theory
DIF: Differential item function
ENDS: Electronic Nicotine Delivery Systems
EV: E-vapor
FDA: Food and Drug Administration
GFI: Goodness of fit index
ICC: Intraclass correlation coefficient
IRB: Institutional review board
ITT: Intentions to Try
ITU: Intentions to Use
ITDU: Intentions to Dual Use
ITS: Intentions to Switch
MRTPA: Modified Risk Tobacco Product Application
PCA: Principal components analysis
PMTA: Premarket Tobacco Product Application
PRO: Patient-reported outcome
RMSEA: Root mean square error of approximation
SD: Standard deviation
SME: Subject matter expert
