# Supplementary material for: Development and validation of behavioral intention measures of an E-vapor product: intention to try, use, dual use, and switch
Source: Health Qual Life Outcomes. 2021 Apr 15;19:123. doi: 10.1186/s12955-021-01764-2 (PMC8048046; doi:10.1186/s12955-021-01764-2)
Supplement: Supplementary file 1 — Additional file 1. Participant Demographic Characteristics Across the Full Sample and 5 Sub-groups. Description of data: Summary of participant demographic characteristics for the full study sample, as well as for the five study sub-groups. [file 12955_2021_1764_MOESM1_ESM.docx]

| Participant Demographic Characteristics Across the Full Sample and 5 Sub-groups | | | | | | |
| --- | --- | --- | --- | --- | --- | --- |
| Demographic Characteristic | Full Sample (N=2,943) | ASPQ (n=508) | ASNPQ (n=648) | EV Users (n=560) | Former Users (n=577) | Never Users (n=650) |
| Gender % |  |  |  |  |  |  |
| Female | 56.2 | 54.5 | 60.3 | 59.1 | 50.3 | 56.0 |
| Male | 43.8 | 45.5 | 39.7 | 40.9 | 49.7 | 44.0 |
| Ethnicity % |  |  |  |  |  |  |
| Non-Hispanic | 92.6 | 92.7 | 95.1 | 90.9 | 92.0 | 92.2 |
| Hispanic | 7.3 | 7.3 | 4.8 | 9.1 | 7.8 | 7.7 |
| Race % |  |  |  |  |  |  |
| White/ Caucasian | 86.2 | 84.6 | 87.2 | 88.2 | 88.4 | 82.8 |
| Black/African American | 8.5 | 11.0 | 7.7 | 7.1 | 7.6 | 9.2 |
| Asian | 2.4 | 2.2 | 1.7 | 3.0 | 1.0 | 4.0 |
| Native Hawaiian/ Pacific Islander | 0.5 | 0.4 | 0.5 | 0.9 | 0.2 | 0.5 |
| American Indian/Alaska Native | 2.6 | 3.1 | 2.8 | 3.4 | 2.4 | 1.4 |
| Other | 3.4 | 3.1 | 3.1 | 3.8 | 3.1 | 3.8 |
| Region % |  |  |  |  |  |  |
| Northeast | 22.9 | 25.0 | 22.2 | 23.6 | 21.0 | 23.2 |
| Midwest | 21.6 | 19.5 | 23.6 | 20.5 | 22.9 | 21.1 |
| South | 31.8 | 33.1 | 34.1 | 29.1 | 31.2 | 31.2 |
| West | 23.2 | 21.9 | 19.9 | 26.1 | 24.4 | 24.0 |
| Age |  |  |  |  |  |  |
| Mean (SD) | 52.5 (14.1) | 51.3 (13.4) | 51.5 (12.6) | 48.4  (13.5) | 58.0 (14.0) | 53.1  (15.3) |
| Range (years) | 18-90 | 21-83 | 22-85 | 18-78 | 18-88 | 18-90 |

Table legend: Summary of participant demographic characteristics for the full study sample, as well as for the five study sub-groups. Raw percentages are reported; therefore, percentages do not always add to 100% due to missing data. ASPQ = adult smoker planning to quit; ASNPQ = adult smoker not planning to quit; EV= e-vapor.
